# In Prototypical Autism, the Genetic Ability to Learn Language Is Triggered by Structured Information, Not Only by Exposure to Oral Language

**DOI:** 10.3390/genes12081112

**Published:** 2021-07-22

**Authors:** Laurent Mottron, Alexia Ostrolenk, David Gagnon

**Affiliations:** 1 Psychiatry and Addictology Department, University of Montreal, 2900 Blvd Edouard-Montpetit, Montreal, QC H3T 1J4, Canada; alexia.ostrolenk@umontreal.ca (A.O.); david.gagnon.7@umontreal.ca (D.G.); 2 CIUSSS-NIM, Riviere-des-Prairies Hospital, 7070 Blvd Perras, Montreal, QC H1E 1A4, Canada

**Keywords:** language, autism, development, perception, veridical mapping, autistic interests, deep phenotypes, clusters/subtypes, neurodevelopment

## Abstract

What does the way that autistic individuals bypass, learn, and eventually master language tell us about humans’ genetically encoded linguistic ability? In this theoretical review, we argue that autistic non-social acquisition of language and autistic savant abilities provide a strong argument for an innate, human-specific orientation towards (and mastery of) complex embedded structures. Autistic non-social language learning may represent a widening of the material processed during development beyond oral language. The structure detection and manipulation and generative production of non-linguistic embedded and chained material (savant abilities in calendar calculation, musical composition, musical interpretation, and three-dimensional drawing) may thus represent an application of such innate mechanisms to non-standard materials. Typical language learning through exposure to the child’s mother tongue may represent but one of many possible achievements of the same capacity. The deviation from typical language development in autism may ultimately allow access to oral language, sometimes in its most elaborate forms, and also explain the possibility of the absence of its development when applied exclusively to non-linguistic structured material. Such an extension of human capacities beyond or in parallel to their usual limits call into question what we consider to be specific or expected in humans and therefore does not necessarily represent a genetic “error”. Regardless of the adaptive success or failure of non-social language learning, it is the duty of science and ethical principles to strive to maintain autism as a human potentiality to further foster our vision of a plural society.

Autism is a neurodevelopmental variant currently defined by the association of reduced or atypical overt socio-communicative behaviours, intense interests, repetitive movements, and perceptual manifestations [1]. Its prevalence is highly dependent on the heterogeneity tolerated by its definition from approximately 1/200 for prototypical autism to 1/50 for the autism spectrum as currently defined [2]. In its prototypical form, autism involves a major delay in the processing and production of oral language and, on the contrary, the preservation of non-verbal intelligence [3,4]. In about half of autistic individuals (depending on the stringency of the prototypical phenotype), it is associated with a series of talents and strengths amongst which the most documented are pattern detection and manipulation [5].

We are at a critical point in advancing our knowledge about the role of genetics in the occurrence of autism. On the one hand, having an autistic condition is highly concordant in monozygotic twins and clinical pictures variably related to this condition demonstrate familial aggregation in siblings [6]. This communicates beyond any doubt that genetics are involved in the etiology of autism. On the other hand, the role of rare variants with a strong effect, essentially *de novo*, explains less than 5% of the overall heritability of autism [7]. It mainly concerns the subgroup of “syndromic” autistics who are often non-verbal and have an intellectual disability, which is poorly representative of the majority of the autism “spectrum” as currently defined (see References [8,9] for the justification of studying distinctly syndromic and non-syndromic autism). Another limitation in our understanding of the contribution of genetics to autism is the variability of the transmitted phenotype. For example, symptom severity can be highly variable between autistic monozygotic twins [6]. The specific form taken by the condition is not transmitted through the same mechanisms as having the condition. This is particularly true for oral language that can range from almost non-existent in non-verbal or minimally verbal autistic people to the highest levels of sophistication as in individuals designated until recently as those with “Asperger’s syndrome”.

The human capacity to detect and then master the language they are exposed in their environment is the very example of gene–environment co-evolution [10]. It allows for variability in the nature of the language learned; there are seven thousand languages on Earth with numerous phonetic and morphosyntactic variations, and even distinct forms of externalization such as sign language. Despite the vast repertoire of languages, the developmental sequence of acquisition for all languages is similar in neurotypical children. This innate ability that characterizes the human species, regarding the learning and then producing oral language after being exposed to it, is atypical in autism. We are therefore at the crossroads between a condition, autism, and a faculty, language acquisition, that are both genetically determined. Each influences the scientific perspective on the other, leading us to consider what autism can teach about the innate nature of language, its mode of acquisition, and use.

In this conceptual article, we will tackle the following question: what, in our conception of the innate human ability to acquire language through participation in social interaction and exposure to oral language, must to be adapted to enable the non-social acquisition of language that characterizes prototypical autism? We will limit our study to non-syndromic phenotypes. We will therefore not discuss the molecular mechanisms involved in the ability to speak and read, nor the effect of *de novo* mutations of “language genes” on language acquisition in autism such as, the most studied of them, Fox P2, which is not involved in autism [11]. Neither will we address the specific effect of the common variants with a weak effect listed by GWAS, which has inventoried hundreds of rarely replicated genes that are minimally informative on their combined activity [12] and thus stumbles on the problem of heterogeneity [13].

## 1. The Nature and Level of Differences in Language Ability among Humankind

The alteration of two central and interrelated human abilities in autism, socialization and language, raises questions about the variability tolerated by the human species [14] (p. 178). We endorse the idea that the constructionist model that stipulates a prerequisite of reciprocal social interaction for autistic language learning has limitations to account for the figures of language acquisition in prototypical autistic people [15]. We further propose a new perspective on how autism could enlighten our understanding of the ability of innate language acquisition: autism and typical development can provide information about each other [16].

As an alternative to considering autism as a genetic “error”, we may consider this human variant as displaying another form of species-specific intelligence, extending for better or worse the realization of initial human possibilities. Atypical learning and processing of complex information in autism provides information on the level of abstraction, complexity, or versatility of what makes us human in terms of information processing. It is the structural properties and the level of complexity, broadly defined, and their successful manipulation by humans that is species-specific. The normocentric constructs forged to account for the material-specific language acquisition of non-autistic children when they are exposed to oral speech (e.g., language acquisition device) may not apply to the same extent to autistic people. Particularly, their “material specificity to oral language” has to be modified [17]. Alternatively, the alteration of a function (e.g., language learning) in autistics is not straightforwardly equated with its deficit. This article aims to unify the learning of language by autistic people and its chronology, communicational peculiarities, relationship with other cognitive systems, and extremes of performance or challenges. We will discuss how language learning as a genetically determined human function may lead to typical (e.g., mastery of language) as well as atypical achievements (e.g., special abilities). Despite its non-conformity to the dominant learning pathways, it may still represent the achievement of the same human function.

## 2. Autistic Learning of Complex Structures: The Veridical Mapping Model as a Structure Acquisition Device

### 2.1. From the Superior Role of Perception in Autism to the Mastery of Generative Structures

Relative (in comparison to their performance in other domains) and in some cases absolute (in comparison to the general population) strengths in autistic people have been reported over the last two decades. More than half of autistic individuals present at least one of these strengths (46% had at least one parent-reported talent and 23% at least one personal strength [18]; 62.5% had isolated skills and 58% had perceptual peaks [19]). The frequency of autistic strengths largely surpasses the more visible phenomenon of autistic “savants”. These strengths involve low-level perceptual over-functioning such as pitch discrimination [20], absolute pitch, pitch memory [21], visual discrimination (here they outperform typical individuals by 30% at the group level [22]), pattern detection and manipulation (particularly of letters even in the absence of oral language [23] for which they detect as much as twice the number of targets that typical children do in certain tasks), musical memory [24], and mental rotation [25], up to and including fluid intelligence in non-verbal abstract reasoning [26].

The enhanced perceptual functioning model (EPF; [27]) proposes that perception in autism demonstrates higher performance, greater involvement, and more autonomy than that observed in the general population. This model is used as an explanatory framework for multiple behavioural, cognitive, and brain imaging variants related to perception and their interaction with the other cognitive functions that characterize autism. Two complementary models have been developed. The veridical mapping model (VM; [28]) reviews the structural homologies shared by the informational material involved in autistic savant abilities, either simple (e.g., absolute pitch) or complex (e.g., calendar calculation). It proposes a mechanism for the developmental and creative extension of autistic special abilities that involve language-like structures such as calendar calculation and those that are essentially non-verbal such as three-dimensional drawing. This will be discussed in the following section of this paper. Finally, the trigger-threshold-target model (TTT; [29]) reviews the neurobiological mechanisms of microcellular plasticity responsible for EPF that will not be addressed here.

### 2.2. Veridical Mapping and Autistic “Special Abilities”

The first principle of the veridical mapping model states that autistic preschoolers spontaneously detect and orient themselves preferentially towards complex structures that exhibit a higher density of isomorphisms—any perceptual structure with a certain structural similarity—than those for which these properties are absent or less rich. According to this hypothesis, it is the multi-scale recursive nature of information to which they are exposed that makes it of interest and triggers the implicit learning of their rules and regularities. This information (pitch scales, colours, numbers, and letters) is presented to the child in highly redundant chains, therefore exhibiting embedded isomorphisms. Written symbols and pitches are also a family of forms that share a configurational resemblance and a high level of within and between-code recurrences. They form larger redundant units at a higher level of organization such as words or musical sentences that share a roughly similar organization/syntactic structure.

A second principle of the VM model is indeed that the autistic brain tends to map isomorphic structures presented in different perceptual modalities onto each other. In the same way that an information format maximizing redundancy triggers an interest and implicit learning, the structural similarity between two layers of perceptual information or “between-code isomorphisms” triggers stable intermodal connectivity between two perceptual expertise regions, possibly favoured by enhanced microstructural plasticity [24]. Indeed, absolute pitch, synesthesia, and early knowledge of letters and numbers involve the stabilization of coupling between homologous elements belonging to an ordered series of auditory and visual information (the name of a note with the note, the name of a letter with a colour or another sensation, or the name of a letter with a grapheme) [30]. Several manifestations of language–perception coupling displaying a VM structure are indeed observed more frequently in autism: absolute pitch (11% of autistic people [31]), synesthesia (up to 20% [32,33]), and hyperlexia (6% [34,35] to 24% of autistic people according to the definition and population studied) [5]. The information involved is generally composed of small perceptually segmented units that present a familial resemblance. The special ability in this case consists of producing an element when presented with another coupled item such as a note and its name for absolute pitch, a letter and a particular colour or sensation for synesthesia, and pronounced words and written code for hyperlexia.

The two VM principles may act concurrently as the choice of the ordered series implies an initial grasp of a structure common to elements with a familial resemblance before their mapping with another homologous element of another hierarchical/ordered sequence [30]. The neuronal stabilization of such intermodal couplings linking together the homologous elements of two isomorphic structures forms the unit on which future autistic “special abilities” are built, mastering complex syntax, and producing complex chains according to the rules of this syntax: certain autistic musicians have perfect pitch, certain calendar calculators present with initial hyperlexia, and synesthesia is common to both.

### 2.3. Savant Syndrome as an Extreme of Autistic Abilities

Certain children of whom a disputed but large majority are autistic develop extreme self-taught abilities involving complex sets of “syntactic” rules such as mastering language heard only through television and/or cracking the codes of calendrical regularities and other examples [36,37]. This has been characterized as “savant syndrome”. Savants generate the flexible use of the units of a sometimes-idiosyncratic domain composed of embedded structures (See Table 1), ultimately manifesting creative mastery of the laws that compose them that is not limited to memorization. A striking example is calendar calculation in which a person who has shown early detection of numbers and letters spontaneously turns to calendars (e.g., [38]). From exposure to only a limited number of calendars, they implicitly understand the laws that govern them. They can then determine the day of the week corresponding to a given date without using an explicit algorithm. Access to this information is bidirectional: the person can produce a list of the years that March begins on a Friday as quickly as they can give the day of the week of 31 July 1914. As discussed in the following, the structural similarity between autistic special abilities and the way autistic people master language may be reciprocally informative.

## 3. The Developmental Sequence of Language Acquisition in Autism

### 3.1. The Apparent Heterogeneity of the Language Phenotype in Autism

Language in autism is atypical in many ways regarding the path and mode of learning, semantics, intonation, thematic content, and pragmatics. These were considered to be diagnostic criteria in the early years of the description of autism in school-age children [39]. Moreover, the reduced intention to communicate and most often the very ability to communicate is intrinsic to the current definition of autism [1].

However, do these atypical characteristics lessen the autistic individual’s aptitude to master the formal aspects of language? Several observations in the half-century following the discovery of this condition led to the dissociation of language skills from socio-communicative atypicalities. The same people who presented marked and prototypical clinical profiles at preschool age and who were minimally verbal could sometimes later correctly use language in adulthood. Therefore, that which was deficient or atypical in these autistics’ use of language did not necessarily concern language competence itself. This finding is consistent with the integration of individuals first described by Asperger into the definition of autism. These individuals master complex language from childhood, whereas as adults they exhibit socialization skills and types of interests similar to those described by Kanner.

How, then, can the same condition be associated with such wide variations in the acquisition, timing, form, and use of language, from silence to exceptional mastery? The operative conclusion to this question in the current diagnostic criteria of autism is that the level of language does not contribute to the identification of autism. Its impairment or its integrity would only modify the presentation and adaptive capacity of autistic people. Alternatively, assimilating language figures in autism to a dimensional, uninformative, quasi-random variation [40] may disregard precious heuristic information on the mechanisms at play in this condition for language, as well as any type of autistic learning mechanism and its unfolding. Language development in autism in its various forms represents an example of the “Wilson effect” [41] in which genetic constraints are more informative for the final development of a function than to its initial presentations.

Consistent with this position, there are three orthogonal variables that structure the autistic language phenotype: the age of speech onset, the presence or absence of an intellectual disability, and the comorbidity with a developmental language disorder (DLD). First, the age of speech onset of people diagnosed with autism displays a bimodal distribution. One subgroup presents with a speech onset delay (SOD) or the early regression/loss of language (or marked transient atypicalities without a delay in a small proportion of them). It corresponds to individuals who are diagnosed early and to the first prototypical description of autism. Another subgroup does not present with SOD. It broadly maps with the former category of Asperger syndrome, now considered in the DSM 5 as autism with typical intelligence and language structure. It generally consists of individuals diagnosed around the end of their preschool years or later. These individuals represent approximately 20% of the overall prevalence of the autism spectrum [42] but their prevalence is increasing based on a current drift of the criteria. A third subgroup contains autistic people with a non-verbal intellectual disability frequently accompanied by identified neurogenetic syndromes that combine the language limitation associated with intellectual deficiency to an autistic language acquisition trajectory (approximately 10–15% of the overall prevalence [7]). Finally, certain autistic children display the same type of language deficits that are found in isolation in non-autistic children with DLD [43]. This subgrouping may oversimplify the clinical reality as certain individuals simultaneously display limitations of several of these functions/groups. Here, we will focus on the first group which may limit the generalization of our model to other subgroups. Prototypical autism combines historical primacy and fast, reliable identification with the most clearly individualized language phenotype [8,9].

### 3.2. Pre-Clinical Phase: Attentional Differences Preceding Recognition of the Syndrome

Autism in its prototypical form is usually detected by parents around the middle of the second year of life through a set of mainly socio-communicative signs (decreased response to name, social synchrony, oriented smiling, initiative and response to joint attention, imitation, and eye contact) and language signs (absence of language development or loss of the few words already acquired by the child) [44,45].

Before autism is visible to a non-expert observer, low and high risk groups are differentiated by the nature of their attention towards social and non-social targets. This has been highlighted by prospective longitudinal studies of siblings of children already diagnosed with autism, one fifth of whom will be diagnosed later. Perceptual signs begin to diverge from typical development at nine months of age on average [46], whereas social signs begin to diverge at 12 months [47]. From the age of 13 months, alterations in these two domains predict the subsequent occurrence of a prototypical autistic presentation additively but independently and sometimes in isolation [48]. Alterations in the two domains precede the objectivation of language delay [49].

A meta-analysis of the non-social signs preceding the diagnosis [50] revealed that children “at risk” for autism (siblings of an autistic child) visually fix on objects for longer durations [51,52] at the end of the first year of life regardless of their future diagnosis. At 12 months, most children who will later be diagnosed with autism present atypical visual exploration [53]. Prolonged target fixation can be seen either as a deficit of disengagement [54] or, on the contrary, as the effect of special capabilities in the detection of targets and visual search, particularly of letters as attested by several convergent studies [23,55,56,57]. The latter is consistent with one third to one half of diagnosed autistic children exhibiting enhanced non-verbal abilities for their chronological age [58]. The manifestations of auditory hypersensitivity (e.g., covering their ears with their hands when exposed to physical noises or a human voice) are maximal during the non-verbal or minimally verbal period [59]. Atypical socio-communicative behaviours and interests are observed before the emergence of formal language even before its atypical development is noticed.

### 3.3. Atypical Language Progression

In the preschool years, the attention of autistic children is directed towards interests that are mostly non-social. This is coherent with the observed plateau of several years without evident progress in the development of social, language, adaptive, and imaginative neurotypical skills [60,61,62]. This is emphasized in the case of oral language when the apparent plateau follows a regression, defined as the loss of previously acquired words after apparent typical development. In these children, the first words are reported by parents to occur earlier than those of typical children but the time period separating the utterance of the first words and two-word sentences is at least double [63]. These autistic children may remain silent for several years or only utter unrecognizable vocalizations idiosyncratic to each child.

The language plateau is not always characterized by complete silence: the absence of communicative language (requests or instances of interactive speech or the use of pragmatic terms) can coexist with “labels” identifying “natural kinds” [64]. The child can thus name aloud the shapes he/she encounters, most often those belonging to an ordered series that he/she is interested in (letters, numbers, colours, geometric shapes, or animals). When these shapes are letters, the situation has been individualized as hyperlexia. In its most extreme forms, the child reads fluently but does not speak (see for example References [65,66,67,68]); such a large discrepancy between reading skills and oral language level was found in 29 of the 82 published cases of hyperlexia [35]. Access to and the understanding of language codes in such cases relies more on non-communicative forms of language than in neurotypical children. This is consistent with the results of a preliminary study suggesting that half of prototypical autistic children with access to screens present an interest in and knowledge of letters and numbers during their third year of life, significantly ahead of general population norms (U.S. Department of Education [69]). For some, letters are learned in a language other than that which is spoken at home (personal observation, LM and AO; see also References [35,70,71].

The progression of expressive language is marked by a succession of atypical language features modulated by chronological age and IQ: immediate echolalia in the presence of a frequently age-appropriate phonology [72], then delayed echolalia, then pronoun reversal, and then stereotyped language [73]. Immediate echolalia is more characteristic of autism when it consists in the verbatim repetition of exchanges between people that are not addressed to the child, now mostly extracted from television/YouTube programs (personal observation, LM). It differs from the echolalia of sentences addressed to the child observed during typical language acquisition and is predominant in non-autistic language disorders. Immediate echolalia testifies to phonological (prosodically contrastive) echolalia [74] that demonstrates its partially communicative and semantic nature or intention.

Delayed echolalia involves sentences that are presented several times in the same way in the environment at some distance from their occurrence such as regarding cartoons, advertisements, or parental instructions. We can sometimes grasp on-the-fly the transformation of stereotyped language into syntactically normal language in the form of mitigated echolalia [75] in which the lessening of deferred echolalia by substitution of one of the original terms by a more adequate term for the current utterance situation occurs. Better understanding is associated with increased mitigation [76]. Stereotypical language is functional but maintains traces of its origin (word order, adult language, and accents) and is heard identically on multiple occasions. In an extension of this phenomenon, in pronoun reversal (when the child asks for a drink by saying “you want water”), the child makes a request by maintaining the personal pronoun used by others to address him/her. Such pronominal reversal represents the last form of delayed echolalia before the mastery of “normal” language. However, it retains a certain level of rigidity, hyper-grammaticality, and formalism [77,78,79] that preserves their diagnostic value in adulthood.

All of these peculiarities are most prevalent around the age of 4 to 6 years at the resumption of language at the end of the plateau and mostly disappear when language normalizes [80]. The plateau can last until the age of 8 [81], 9 [82], or 11 [83]. The late emergence of language is also well documented in autism as shown by Picket et al. [84] who identified 167 autistic children who acquired language after five years of age in a conservative review. Anecdotal cases of language recovery in adulthood have also been reported [85]. The slope of acquisition once language progression has restarted is generally steep [82,86]. Kanner [87] noticed that non-verbal autistic children surprised their parents in emergency situations when they uttered grammatically correct sentences, suggesting that they had accumulated a large amount of language information before their first use of speech. Two studies [88,89] reported the sudden emergence of fully formed speech after the apparition of reading skills in several subjects. The extent of oral language recovery has been associated with the level of non-verbal IQ [63,82,86].

Meta-analyses on the effect of interventions on language recovery, whether it is before five years of age or later, demonstrate that they have only a minimal influence on the language development of these children [90,91,92]. Approximately 7% of autistic children naturally demonstrate a quick catching-up of their language skills, developing from a generally delayed language ability to the expected language level for their age [82,86]. When it appears, language is essentially self-taught [15] and autonomous access to written or web-based information could play a major role in its emergence [35,70,71]. Although nonverbal IQ remains the main predictor of ultimate language access, it does not guarantee it [93]. A quarter [94,95] to a half [96] of autistic children under the age of 10 are considered to be mute or minimally verbal. The notion of non-verbal autism must indeed be viewed critically as its definition is uncertain [97].

In summary, the maturation of oral language in prototypical autistic children follows a “bayonet-shaped” sequence composed of three steps: (a) an almost normal start but with an atypical imbalance of interests and attention for auditory and verbal stimuli; (b) a nonverbal or minimally verbal plateau with or without the loss of the first words; and (c) atypical rapid resumption resulting most often in syntactically correct language after six years of age [63]. In a substantial proportion of cases, this sequence is coupled with an early interest in written code with decoding skills superior to comprehension in the hyperlexic period. In most cases, it predicts the catching-up in reading comprehension after five years of age [98]. The nature of the oral utterances or reading prerequisites that a child demonstrates during this sequence supports the idea that oral and written language are not primarily learned in a communicative way in autism. This mode of self-taught learning and the cracking the code of complex structured information to which they are exposed to moves beyond language learning and finds its extreme expression in autistic “savant” abilities as we will now see.

### 3.4. Special Skills and Language Follow Similar Development Paths in Autism

There are deep similarities between the ways autistic people develop a special ability and their sequence of language acquisition (see Table 1). Most intense interests and autistic special abilities contain a language element [99]. In autism, both language and autistic special abilities are self-taught. Language can develop without the evidence of social reinforcement [15]). Such learning is mainly implicit and favours large scale isomorphic units as shown by the delayed echolalia of sentences presented in a fixed format. Both involve language in the visual modality (letter recognition) earlier or at a higher level than in typical children but they are associated with a language delay in a large fraction of the autistic population. Finally, numerous savants present both hyperlexic and another savant ability [38,100].

We propose that exposure to structured material including but not limited to language “triggers” both the development of special abilities and language in autism. However, such language needs to be presented in a more isomorphic manner and with a higher level of structural recurrence (written language, calendars, YouTube nursery rhymes, etc.) than the oral language of their social environment that is of much more importance for typical children. More importantly, the detection-coupling-syntax developmental scheme may be the same for language and special abilities. Both cases include the following steps: (a) the detection of structural laws in the chosen material comprised of identical units constituting larger structures; (b) the mapping of units from one modality to their counterpart in another modality; (c) the stabilization of the coupling between the homologous elements; and (d) the generative mastery of a code with several embedded levels, allowing the production of new well-formed utterances.

In short, the heterogeneity of the fields that scholarly capacities are applied to in autism and their structural similarity allows for abstracting the principles at stake beyond the domain of the information involved. Language learning and special abilities in autism may arise from the same mechanism and competence provided that the class of objects to which they apply is widened. However, this difference does not alter the fundamental principles that are embedded in human genetic code, thus ruling them. These principles are also those that govern language learning in typical children as we shall observe.

### 3.5. The Comparative Approach between Autistic Language and Chomskyan Nativism

Although their nature is still a subject of debate, there are genetic constraints to the orientation towards oral language and its acquisition. These include the “innate biases and strategies that place constraints on perception and learning” that “set the parameters for a particular pattern from among those innately provided” [101]. In Chomskyan nativism [102], the faculty of language narrow sense (FLN), includes the core computational mechanisms of recursion that allow syntax generativity and its mapping to perceptual and motor interfaces. The acquisition of language is dependent on an ability to recognize language, detect a recursive structure in it, and adapt to one’s mother tongue. The high level of abstraction of syntactic universals is revealed in a comparative approach, allowing common and different aspects to emerge between the acquisition of various syntactic structures by humans.

Just as the comparison of human languages defines the level of abstraction of the universals of acquisition and structure specific to human languages, comparison of various special autistic abilities provides information about their development and common neurocognitive mechanisms. We support Kissine’s [15] statement that autism can help determine the constraints of the level of abstraction necessary for the extraction of language structure and language acquisition in humans. In addition to a simple comparative approach between different realizations of the same ability, this requires a meta-comparison between the plurality of typical language achievements and the plurality of special autistic abilities that may rely on the same core aptitudes or mechanisms. The meta-comparison between the autistic and non-autistic deployment of special abilities and language potentiality reveal how both manifest a common, human property of language generativity and the mastery of recursive structures, respectively. We are therefore at odds with the conclusions of Pinker [103] (p. 62) who wrote “Together with robots and chimpanzees, people with autism remind us that cultural learning is possible only because neurologically normal people have innate equipment to accomplish it”, and Tomasello [104] (p. 689) who wrote “neither apes nor children with autism have—at least not to the same extent as typically developing human children—the motivation or capacity to share things psychologically with others. This means that they both have very limited skills for creating things culturally with other persons”. For them, the triangular comparison of typical humans, apes, and autism results in the rejection of autism outside of species-specific identity.

The emergence of autistic special abilities follows exposure to structured material that shares commonalities with language. The formal properties and our improved understanding of the materials on which autistic intense interests are directed towards and that permit the development of special abilities reveal common mechanisms and constraints with language. For example, each of the two components of multimodal units such as perfect pitch, synesthesia, letter denomination, or calendar calculation all belong to (a) finite families of (b) highly similar shapes each time they occur, (c) presented spatially or sequentially in real terms and (d) structured by complex but recurring constraints (or within/between code isomorphisms). Such invariance suggests that all of these characteristics, albeit quite minimal, are sufficient to trigger an interest or orientation and be the subject of subsequent mastery including language generativity. The veridical mapping framework implies that the abilities required for language acquisition such as the mastery of recursive structures are initially used and developed in other spheres. These are related to autistic children’s interests and will subsequently be useful or reused for linguistic decoding. Language progression would then be delayed and language decoding would therefore be less dependent on social cues and rely more on the structure of the exposed material [70]. It should therefore not be surprising that the acquisition of language is atypical in its timeline, peculiarities, and rigidities.

## 4. Material and Species Specificity

### 4.1. Cortical Reallocation and Idiosyncrasy in Autism in Language-Related Tasks

Language in autism can be understood from the perspective of the heterogeneity of autistic language phenotypes from impaired to exceptional and the steps of its development [28]. Brain activity associated with oral language is at first abnormally low and then atypically localized. Children with a genetic predisposition to autism display a hyper-connected auditory–motor association cortex at nine months but also a low specialization for voice [105]. Importantly, around the age of language acquisition, autistic people demonstrate enhanced auditory–visual connectivity within the language regions and between them and the visual cortex, consistent with the audio–visual advances observed in multimodal coupling [106]. These alterations together with a reduction in the lateralization of activation when exposed to oral speech are among those that predict an autism diagnosis at 24 months [107]. This pattern can still be observed in adulthood [108,109]. This differentiates between autistic adults with and without an initial speech onset delay who present with considerable differences in cortical allocation [110,111]. The variability of cerebral allocation in areas of perceptual expertise can therefore be observed both at the individual and group level. When exposed to speech-like material, autistic adults who had a SOD exhibit overactivation of the Heschl gyrus and the area responsible for the perceptual part of language processing [111]. Alternatively, autistic adults who did not have a language delay exhibit activation of a large area that includes the brain cortex. This area is activated in neurotypicals when processing language linguistically and even extends to motor cortices [111].

In the visual modality, there is also greater variability in brain expertise regions. The relative weighting of the activation [112,113] and connectivity [114,115] of perceptual vs. language networks in linguistic tasks deviates towards domain-specific visual expertise. This has been verified in language tasks [116] and reasoning tasks [26] in which such hyper-activation is associated with faster performance than typical individuals of the same intelligence demonstrate [26]. Conversely, the localization of activation observed during the information processing that leads to circumscribed localization in typical people is both different from that of typical people when averaged and more different between autistic individuals than between typical people [117]. Overall, this suggests widening of the genetic constraints of the neural and functional allocation of expertise regions, particularly those devoted to language processing.

### 4.2. Autism Involves the Widening of a Genetic Constraint toward Language Material-Specificity

The variations of brain activity observed between autistic and non-autistic people during linguistic tasks predominate in regions that developed the latest in evolution [118,119] and that maintain cross-modal plasticity during early deprivation of perceptual input [29]. Conversely, the widening of the material-specific constraints observed in autism during oral language acquisition suggests an alteration of the mechanism responsible for the orientation toward and selection of the material producing the first linguistic units in neurotypicals. Therefore, we understand development related to language (and complex information in general) in autistic people as the enlargement of the typical language-specific coupling between brain specialization and language.

We propose that inborn linguistic parsing mechanisms preferentially orient autistic people toward any material with “embedded structures”—what we have called a high density of isomorphisms—instead of primarily oral communicative language as in typical people. This extension would apply to any material possessing these properties, possibly to the detriment of oral language. This model is suggested by (a) the atypical attention parameters and behaviours that precede the developmental break or language regression and plateau; (b) the abstract similitude of the autistic and non-autistic sequence of integration of embedded structures; and (c) the enhanced variability of functional and anatomical allocation of the regions involved in the development and use of language in autistic people.

Variability in dominant material-specific constraints or atypical coupling between a specific material and a brain function is the rule and not the exception in phylogenesis as in individual development. They are found at the individual level following the absence of auditory or visual input (e.g., cross-modal plasticity in early visual impairment [120]) and at the population scale in the recycling of the Visual Word Form Area [121]) at the evolutionary level. They have been conceptualized with certain differences as neural reuse [122] or exaptation [123] and were involved in the evolution of human language. Their latest manifestation is the existence of a neural allocation for written code. When the coupling between information processing units and information present in the living environment opens up as in reading, this is “cultural reuse” [124].

The material-specificity for an area of intense interest and the subsequent savant ability are determined by exposure to certain materials and follow prolonged inspection/analysis. Just as there is a critical period for learning the mother tongue for a typical child in the early preschool years, the dedication of a period focused on non-social structured material within a certain modality will partially “freeze” the autistic domain of interest. Savant abilities may, however, expand toward other domains of structured material, although such expansion is constrained by an isomorphic relationship between domains such as for special computational abilities or between calendar and tonal structure [100].

### 4.3. Does Autistic Language Development Fall Outside of Species-Specific Characteristics?

We separate the human specificity in dealing with complex structures—the potential material-specificity for language—from the relative domain-specificity of these mechanisms once they have become established for a particular individual speaking a particular language. We distinguish the material-specificity (the linguistic nature or not of what triggers the processes of which typical language learning would be a special case) from the domain-specificity inherent to the processing of complex information once such learning has taken place. The specialization of a language for a neurotypical group and specialization of an interest for an autistic person in particular would therefore be phenomena of the same nature, resulting from the same species-specific genetic constraints (Anderson and Penner-Wilger [125]).

The enlargement of the scope of information preferentially processed during infancy in autism does not imply a non-human or super-human autistic nature. Privileging written language, calendars, or visual structures suggests an orientation towards materials that maximize the density of isomorphisms such as those found in human language. Moreover, most autistic people usually develop to speaking and a certain number of them speak earlier and better that the typical population. Beyond the strong similarity between typical language acquisition and autistic special abilities, the level and nature of autistic intelligence confirms that autism belongs to what is most human in humans. A quarter of the siblings of autistic children who therefore share the same predisposition to autism as their autistic relative are two standard deviations ahead of the Mullen norms of the non-autistic population at the same age. Forty percent of them have a higher level of non-verbal intelligence than the general population [58]. A previous study demonstrated that when a group of autistic adults were matched to typical adults for global IQ, the autistic group demonstrated a much higher intelligence as measured by non-verbal reasoning than by a verbal intelligence measure [93]. The common low-effect genetic variants associated with prototypical autism are also those associated with intelligence in typical people [126,127]. The level of “severity” and the verbal or non-verbal status of an autistic person is independent of this genetic predisposition to autism [128,129]. Prototypical autism is thus another combination of verbal and non-verbal intelligence and not an intellectual disability.

Access to language in autism with its divergences and convergences with predominant paths results from a deep identity with the access to language that constitutes humanity. Autism consists of the opening of the potentialities of detection, learning, and manipulation of complex human information to families of non-socially determined forms and structures. Following the findings of K. Lorenz, it has been recognized that the relative absence of fixed material-specific instinctual sequences goes hand-in-hand with intelligence; crows are smarter than cranes. The extension of the material processed by a genetically transmitted function and its cultural stabilization is undoubtedly a source of human progress as long as such diversity is welcome in the case of autistic people. Neural reuse (or recycling/exaptation) is the norm and not the exception in evolution [123,125]. These apparent inflections in adaptation are involved in what makes humans what they are specifically in terms of the invention and use of language and the ability to read within language. Emphasizing the reduced dependence of autistic people on social triggers in language development, Pinker and Tomasello’s major argument in favour of their position toward autism considers the most species-specific trait in humans to be their least species-specific trait: their gregariousness.

It is necessary to alter the conception of autism as a genetic “error” in favour of an abstraction of the species-specific constraints and abilities to integrate and potentially master the complex information they are exposed to. Such accomplishments occur by an original path that may explain both the success and challenges of autism. The decisions taken by the non-autistic majority of humans through scientific models of autistic differences (variants in prototypical autism vs. deleterious mutation in syndromic autism), the anthropological status of these differences (contributory to humanity or a disease to be cured), the delineation between autism and non-autism (categorical or dimensional), and their ethical and societal consequences (to cure or to integrate) are intricate and influence each other. The medical model of autism that defines autism as a genetic “error” to be cured, grounded on a partial interpretation of its genetic component, appears to be a recurrent temptation. It is sometimes based on stretched analogies such as using mouse-based behaviourist intervention programs as a model for autistic learning. Alternatively, it may also find its rationale in the most sophisticated phylogenetic considerations such as fine-grained studies of what differentiates humans from apes in reasoning and communication. In both cases, we see the medical model of autism as ethically outdated, scientifically short-sighted, and heuristically sterile.

### 4.4. Recognizing the Human Genetic Nature of Autistic Language Learning Changes Our Strategy for Intervention

The notion of non-communicative language learning does not imply fostering mere passive exposure to information of this nature without interaction with peers. The aim is to make this information accessible [130] to the child and display it in front of him, for example, by using “lateral tutorship”, minimizing typical socio-communicative interaction patterns [88]. We thus take the model of exposure to a mother tongue for the typical child but modify the oral and interactive aspects [35,70,71]. The autistic child is minimally interested in a direct guardianship interaction but is an attentive observer and deferred imitator of what he observes. It is thus advisable to make such information work in front of him while integrating it but without asking for his active participation as manifested by the same behavioural marker criteria as for a typical child. By re-attributing the genetically innate capacity to manipulate and extract complex integrated structures to autism rather than identifying it with a deficit to be treated and to use social cues, we will discover a new rationale to guide our intervention strategies. Access to literacy phone/tablet applications that children learn to use on their own or because they have seen them used in front of them is thus a promising avenue. The entry into oral language via the written code through the manipulation (without specific instructions) of online applications by the child therefore represents a research theme to prioritize.

## Figures and Tables

**Table 1:** Common unfolding of the non-autistic acquisition of oral language and autistic savant abilities.

Competence	Unity Of Information	Recursivity	Embedded Structure	Triggering Exposure	Veridical Mapping	Generativity	Associated Behaviours	Imprinting Effect
**Non-autistic speech**	Syllables	Embedded sentences	Syntax	Oral mothertongue	Language–world referential mapping	Producing new well-formed sentences	Imitation, joint attention, and reference	Irreversibility of Mother tongue
**Autistic** **hyperlexia**	Letters and numbers	Letters embedded in words and sentence chains	Written code	Combined written and oral signs	Isomorphic graphic–phonic structures	Reading unknown words	Orientation toward written code	Restricted/intense interest for printed material
**Verbal savant ability (e.g., calendrical calculation)**	Dates and calendar lexicon	Letters and numbers embedded in month-day-year calendars	Calendrical structure	Calendars	Isomorphic calendar structures	Producing non-memorized dates	Intense interest for dates	Restricted/intense interest for calendars
**Visuo–spatial savant ability** **(e.g., 3D drawing)**	Geons	Micro 3D scale embedded in 3D macro scale	Mechanical structure	3D objects	Isomorphic visual–graphic structures	Producing graphic 3D transformations	Lining up; prolonged visual inspections	Restricted/intense interest for 3D patterns
**Musical savant ability (e.g., memorizing, interpreting, composing musical pieces)**	Pitches	Pitches embedded in musical sentences and pieces	Musical structure	Musical pieces	Isomorphic musical patterns	Composing new musical sentences	Auditory searchesand avoidance	Restricted/intense interest for music
